# Unveiling diversity and adaptations of the wild tomato Microbiome in their center of origin in the Ecuadorian Andes

**DOI:** 10.1038/s41598-025-05816-1

**Published:** 2025-07-01

**Authors:** Stalin Sarango Flores, Viviane Cordovez, Luisa M. Arias Giraldo, Antonio Leon-Reyes, Pieter van ’t Hof, Jos M. Raaijmakers, Ben O. Oyserman

**Affiliations:** 1 Department of Microbial Ecology, Netherlands Institute of Ecology, Wageningen, 6708PB The Netherlands; 2 Institute of Biology, Leiden University, Leiden, 2333BE The Netherlands; 3 Colegio de Ciencias Biológicas y Ambientales, Universidad San Francisco de Quito USFQ, Quito, 170901 Ecuador; 4 Laboratorio de Biotecnología Agrícola y de Alimentos, Ingeniería en Agronomía, Colegio de Ciencias e Ingenierías El Politécnico, Universidad San Francisco de Quito USFQ, Campus Cumbayá, Quito, 170901 Ecuador; 5 Instituto de Microbiología, Colegio de Ciencias Biológicas y Ambientales, Universidad San Francisco de Quito USFQ, Quito, 170901 Ecuador

**Keywords:** Rhizosphere Microbiome, *S. pimpinellifolium*, Center-of-origin, Enterobacteriaceae, Metagenomics, Biological techniques, Ecology, Microbiology, Plant sciences, Ecology, Environmental sciences

## Abstract

Microbiome assembly has been studied for many plant species and is recognized as a key driver of plant growth and plant tolerance to (a)biotic stresses. To date, assembly of the tomato rhizosphere microbiome has been investigated primarily for commercial varieties and field soils subjected to agricultural management practices, whereas the microbiome of wild tomato genotypes in their native habitats remains largely unexplored. This research focused on distinct populations of *Solanum pimpinellifolium* in three natural habitats in the Ecuadorian Andes to identify the taxonomic and functional diversity of their rhizosphere microbiome. The results showed that, despite genotypic differences among the wild tomato populations, different soil types and soil microbiome compositions, the rhizosphere microbiome showed strikingly compositional similarity across the three habitats. Proteobacteria, in particular taxa classified as Enterobacteriaceae, and specific unclassified fungal taxa were highly represented in the rhizosphere of *S. pimpinellifolum*. Metagenomic analyses suggested that the prevalence of Enterobacteriaceae on wild tomato roots may be explained by several traits, in particular nutrient competition, motility, iron acquisition, membrane transport, stress response, and plant hormone biosynthesis. These results reveal a conserved microbiome signature associated with wild tomato rhizosphere in their center of origin. Just as the genomes of wild crop ancestors provide a valuable source of beneficial traits for breeding cultivated varieties, exploring their microbiome in native environments could uncover microbial taxa and traits that similarly contribute to crop growth and health.

**Supplementary Information:**

The online version contains supplementary material available at 10.1038/s41598-025-05816-1.

## Introduction

Tomato holds significant importance for global food security and human health because of its high nutritional value. The incredible domestication journey of tomato through both space and time has left an impact on its genome and microbiome^1–4^. Hence, the genome of wild tomatoes has been used as valuable source of plant genetic traits for crop improvement strategies^5–9^. Similarly, wild tomato growing in its native habitat is a largely unexplored resource for the discovery of beneficial microorganisms that were depleted or lost during domestication. Native habitats can serve as repositories of a high microbial diversity, potentially reflecting long-term associations linked to the plant’s ecological niche and adaptation to specific, even harsh, environmental conditions. However, such beneficial native associations may have become diminished or unnecessary under modern agricultural settings^10^. This is the case with the wild tomato, *Solanum pimpinellifolium*, the closest relative to domesticated tomato, which grows naturally in the semi-arid regions of southern Ecuador, where it has adapted to high daytime temperatures, water scarcity, and other (a)biotic stresses^3,11–13^. Hence, microbial communities that co-evolved associations under such native selective conditions with wild tomatoes may play pivotal roles for the plants’ survival^14,15^.

Consequently, there is a growing interest in unraveling the effects of plant domestication on microbiome assembly in the rhizosphere, the narrow zone of soil surrounding and influenced by the plant roots^16–19^. The rhizosphere microbiome plays an important role in nutrient cycling and plant health^20–23^. Therefore, understanding the taxonomic and functional diversity of the rhizosphere microbiome of wild tomatoes in their native habitats will be essential to pinpoint microbial traits that co-evolved with its host and that may have been depleted or lost through domestication and subsequent breeding. In turn, these ‘missing’ microorganisms or their beneficial traits could be a valuable resource for making domesticated tomato more resilient to (a)biotic stresses.

Here, we collected rhizosphere samples of three populations of wild tomato *S. pimpinellifolium* growing naturally in the Andean Mountain range in southern Ecuador, an area that is part of the species’ center of origin. We reveal that these wild tomatoes were able to assemble taxonomically similar rhizosphere bacteriomes despite growing in different regions, varying soil types with variable microbiomes, even for phylogenetically different wild tomato populations. In particular, Enterobacteriaceae dominated the rhizosphere bacteriome of *S. pimpinellifolium*. Results of the metagenomic analyses further suggest that this dominance may be due to specific traits associated with high competitivity and colonization efficiency. Collectively, these findings improve our fundamental knowledge about the root microbiome of wild crop relatives grown in their native habitats and pinpoint specific microbiome members that may enhance resilience of the domesticated crop cultivars to (a)biotic stress factors.

## Results

### Genetic diversity and genomic relationships of wild tomato populations

**Fig. 1:**
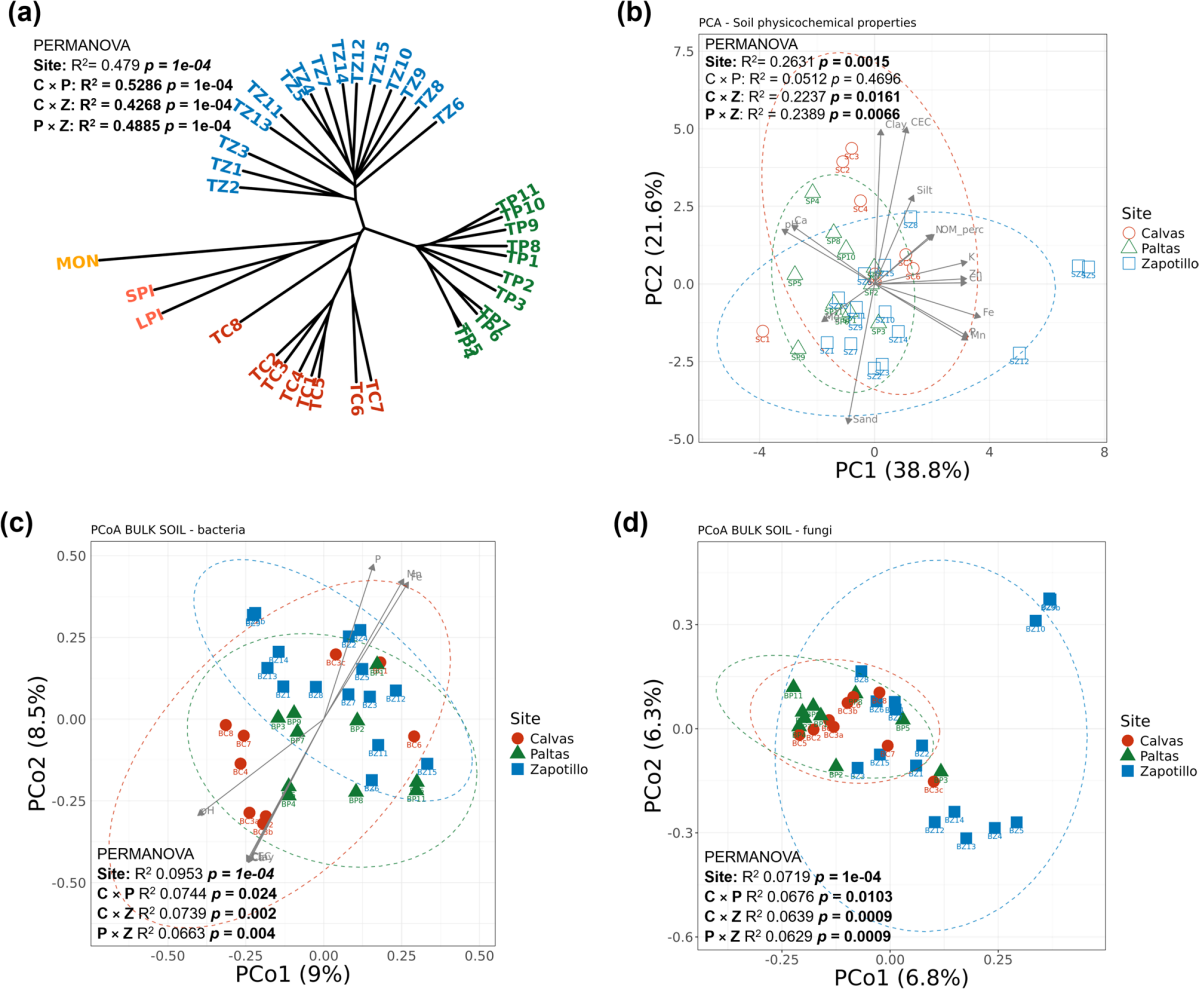
Genetic diversity of wild tomato plants, variation in soil properties and soil microbial communities across sampling sites.** (a)** Genetic diversity of 34 wild tomato samples collected from Calvas (TC), Paltas (TP), and Zapotillo (TZ) based on DArT SNP analysis; the wild tomato accessions *S. pimpinellifolium* (SPI, LPI) and the domesticated tomato *S. lycopersicum* cv. Moneymaker (MON) are included as references **(b)** Principal Component Analysis (PCA) of soil physicochemical properties based on the Euclidean distance across the three sampling sites; **(c)** Principal Coordinates Analysis (PCoA) of bulk soil bacterial communities based on the Bray–Curtis distance between the three sampling sites and their correlation with soil physicochemical properties; **(d)** PCoA of bulk soil fungal communities based on the Bray–Curtis distance between the three sampling sites. Labels BC, BP and BZ refer to bulk soil from Calvas, Paltas, Zapotillo sites, respectively. For each pairwise comparison between sampling sites (C, P, Z), statistically significant differences based on PERMANOVA are indicated.

DArT-SNP genotyping of the 37 tomato samples, including 34 wild tomato plants, two accessions of *S. pimpinellifolium* (LPI and SPI), and one accession of domesticated *S. lycopersicum* cv. Moneymaker (MON), resulted in five genotypic clusters consistent with the three sampling sites (Calvas, Paltas, Zapotillo – three clusters), LPI and SPI accessions (one cluster) and MON accession (one cluster) (Fig. 1a). Furthermore, the genetic diversity of the 34 wild tomato samples was significantly different among the three sites (PERMANOVA, R^2^ = 0.4790, *p* = 1e-4).

## Soil physicochemical properties and bulk soil Microbiome composition

The physicochemical properties of the native soils were significantly different between the sampling sites (PERMANOVA, R^2^ = 0.2631, *p* 0.0015). However, soils from Calvas and Paltas showed similar physicochemical properties (*p* = 0.4696). The first principal component showed that soils from Calvas and Paltas were characterized by higher pH, Ca and Mg, while Zapotillo soils were characterized by higher content of Fe, Mn, P, K, Cu and Zn. The second principal component was represented mainly by soil texture, with soils from Calvas and Paltas, in general, characterized by higher clay, silt and CEC values whereas soils from Zapotillo were characterized by higher content of sand (Fig. 1b; Supplementary Table S3).

No statistically significant differences were found for the alpha diversity (Shannon index) of the bacterial communities in the soils from the different sites (ANOVA, *p =* 0.865), but significant differences were found for the fungal communities (ANOVA, *p =* 0.0347). More specifically, a lower alpha diversity was found for the fungal communities in soils from Paltas compared to soils from Calvas and Zapotillo. Simpson index confirmed a similar trend for bacterial communities (ANOVA, *p* = 0.983) and fungal communities (ANOVA, *p* = 0.0033), with all sites showing relatively comparable values, indicating similar community dominance (Supplementary Figure S5; Supplementary Table S4). The beta diversity was significantly different among the sites for both bacterial (PERMANOVA, *p* = 1e-4, R^2^ = 0.0953; Fig. 1c) and fungal communities (PERMANOVA, *p* = 1e-4, R^2^ = 0.072; Fig. 1d; Supplementary Table S5). In addition, significant relationships were found between physicochemical soil properties and bacterial diversity in native soils (Mantel test, ρ = 0.1973, *p* = 0.0256), but not between the soil properties and the fungal community diversity (Mantel test, ρ = 0.0181, *p* = 0.4081). Seven out of 15 physicochemical soil properties correlated with the bacterial community distribution in the different native soils. These included Ca (R^2^ = 0.35), clay (R^2^ = 0.30), CEC (R^2^ = 0.29) and pH (R^2^ = 0.30) for soils from Calvas and Paltas, and Fe (R^2^ = 0.33), Mn (R^2^ = 0.24) and P (R^2^ = 0.24) for soils from Zapotillo (Fig. 1c).

## Wild tomatoes assemble similar rhizosphere bacteriomes across native sites

**Fig. 2:**
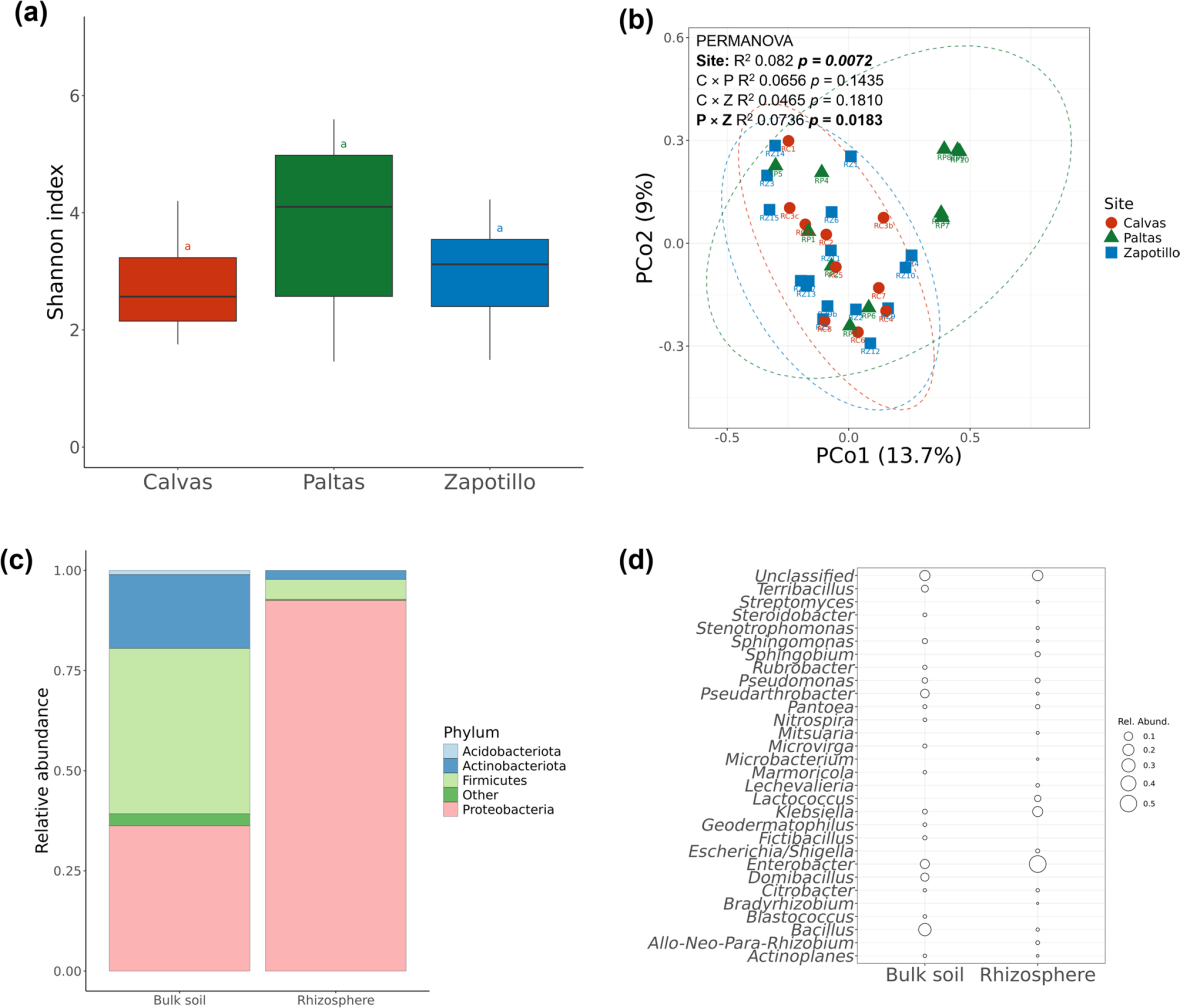
Bacterial diversity in wild tomato rhizosphere.** (a)** Alpha diversity of the bacterial communities in wild tomato rhizosphere based on Shannon diversity index according to each sampling site (Calvas *n* = 8; Paltas *n* = 11; Zapotillo *n* = 15); no significant differences were found by Tukey HSD test *p* < 0.05; **(b)** Principal Coordinates Analysis (PCoA) of bacterial communities based on the Bray–Curtis distance between rhizospheres of the three sampling sites; for each pairwise comparison between sampling sites statistically significant differences based on PERMANOVA are indicated; **(c)** Relative abundance of bacterial phyla in bulk soil and rhizosphere of wild tomato; “Other” category corresponds to grouped phyla with relative abundance < 0.01; **(d)** Relative abundance of highest abundant (top 20) genera found in bulk soil and rhizosphere of wild tomato. ASVs with significant differential abundance between bulk soil and rhizosphere were grouped according their phylum or genera and plotted as stacked bar and bubble charts, respectively.

Bacterial communities differed between bulk soil and rhizosphere (PERMANOVA, *p* = 1e-4, R^2^ = 0.094) (Supplementary Figure S6a; Supplementary Table S5). The alpha diversity of the bacterial community of the rhizosphere of wild tomato, exemplified by the Shannon(ANOVA, *p* = 0.0526) and Simpson (ANOVA, *p* = 0.545) indices, was similar for each of the three sampling sites (Fig. 2a; Supplementary Table S4). Despite significant differences between the bulk soil bacterial communities of the three sampling sites, wild tomato rhizosphere bacterial communities were mostly similar across the sites based on Bray*–*Curtis distance, except for the rhizosphere bacteriomes of wild tomatoes from Paltas (P) and Zapotillo (Z) (PERMANOVA, *p* = 0.0183, R^2^ = 0.074) (Fig. 2b; Supplementary Table S5). For the three sites combined, no significant relationships were found between the wild tomato genetic diversity and rhizobacterial abundance (Mantel test, ρ = −0.1202, *p* = 0.9184) nor between physicochemical soil properties and rhizobacterial abundance (Mantel test, ρ = 0.0582, *p* = 0.2798). For the sampling sites separately, significant correlations were found between specific soil properties and rhizobacterial community composition for Paltas (Ca: R^2^ = 0.23, *p* = 0.0478) and for Zapotillo (Cu: R^2^ = 0.30, *p* = 0.0046) (Supplementary Figure S7).

Differential abundance analysis of the bacterial community composition of the pooled bulk soils and tomato rhizosphere samples identified 197 ASVs as significantly different, i.e., 111 were more abundant in bulk soil and 86 more abundant in the tomato rhizosphere. In bulk soil, the majority of the ASVs belonged to the Firmicutes (41%), Proteobacteria (36%) and Actinobacteriota (18%). In the rhizosphere of wild tomato, abundant ASVs mainly belonged to Proteobacteria (92%), Firmicutes (5%) and Actinobacteriota (2%) (Fig. 2c). At the genus level, the rhizosphere of wild tomatoes was characterized by a higher relative abundance of *Enterobacter*,* Klebsiella*,* Sphingobium*,* Escherichia/Shigella*,* Allorhizobium-Neorhizobium-Pararhizobium-Rhizobium*,* Lactococcus*, and *Lechevalieria* (Fig. 2d).

## Wild tomatoes assemble distinct rhizosphere mycobiomes across native sites

**Fig. 3:**
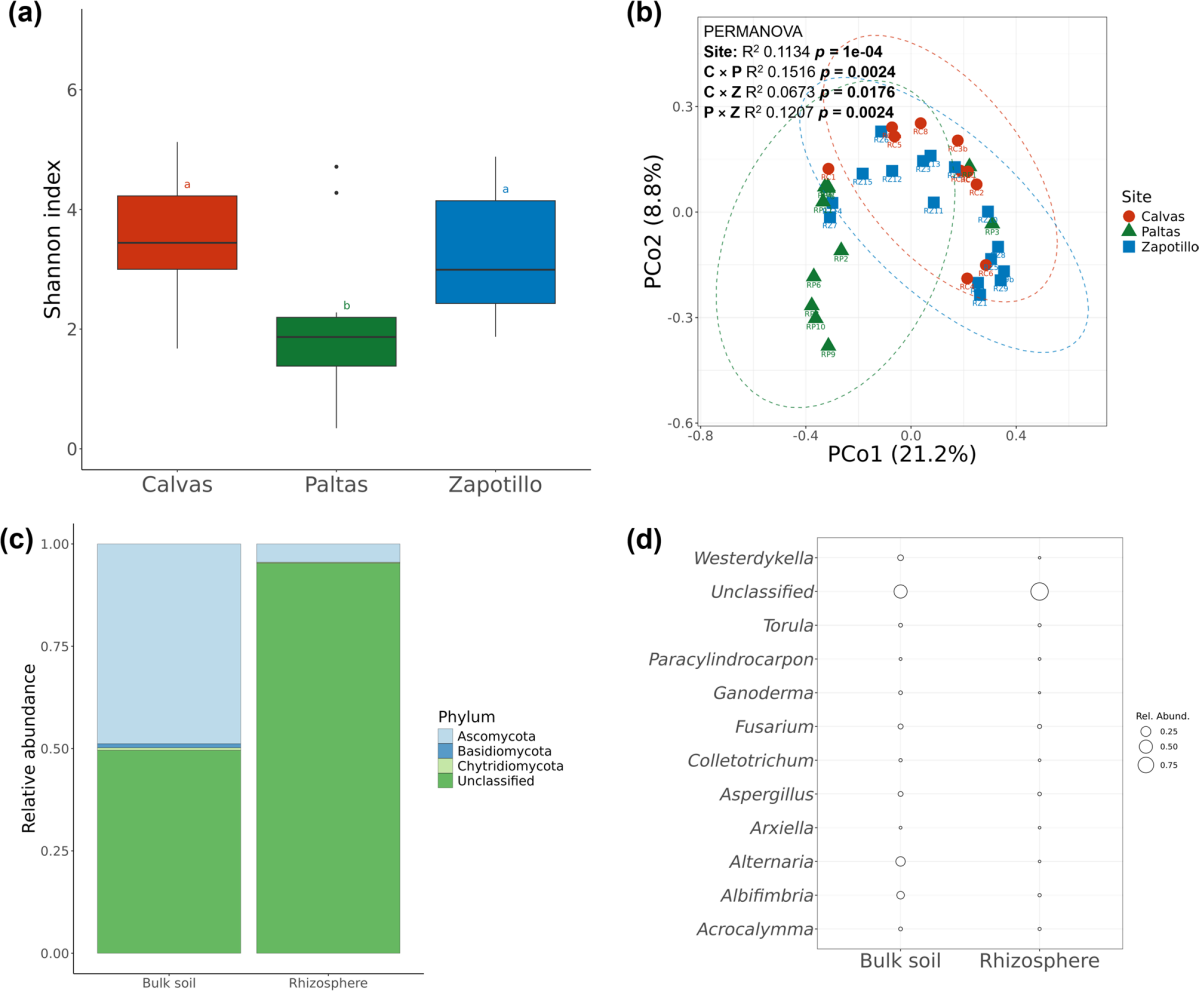
Fungal diversity in wild tomato rhizosphere.** (a)** Alpha diversity of the fungal communities in wild tomato rhizosphere based on Shannon diversity index according to each sampling site (Calvas *n* = 8; Paltas *n* = 11; Zapotillo *n* = 15); different letters above boxplots show significant difference by Tukey HSD test (*p* < 0.05); **(b)** Principal Coordinates Analysis (PCoA) of fungal communities based on the Bray–Curtis distance between rhizospheres of the three sampling sites; for each pairwise comparison between sampling sites statistically significant differences based on PERMANOVA are indicated; **(c)** Relative abundance of fungal phyla in bulk soil and rhizosphere of wild tomato; **(d)** Relative abundance of major fungal genera in bulk soil and rhizosphere of wild tomato. ASVs with significant differential abundance between bulk soil and rhizosphere were grouped according their phylum or genera and plotted as stacked bar and bubble charts, respectively.

The rhizosphere mycobiome was significantly different between Paltas and the other two sampling sites, Calvas and Zapotillo, based on the Shannon (ANOVA, *p* = 0.0094) and Simpson (ANOVA, *p* = 0.0003) diversity indices (Fig. 3a; Supplementary Table S4). Both indices showed lower values at Paltas, indicating higher community dominance by a few ASVs compared to Calvas and Zapotillo. The beta diversity of fungal communities differed between bulk soil and rhizosphere (PERMANOVA, R^2^ = 0.098, *p* = 1e-4) (Supplementary Figure S6b), as well as between the three sampling sites (PERMANOVA, R^2^ = 0.1134, *p* = 1e-4) (Fig. 3b; Supplementary Table S5). Furthermore, a significant relationship was observed between the tomato genetic diversity and rhizosphere fungal abundance (Mantel test, ρ = 0.1936, *p* = 0.0093), but not between the physicochemical soil properties and rhizosphere fungal abundance (Mantel test, ρ = −0.0217, *p* = 0.4665).

Differential abundance analysis of the fungal community composition of the pooled bulk soils and tomato rhizosphere samples revealed 18 ASVs significantly different, 6 ASVs for the bulk soil and 12 for the tomato rhizosphere. The more abundant ASVs in the bulk soil were unclassified fungi (49.6%), Ascomycota (48.8%), Basidiomycota (1%) and Chytridiomycota (0.6%) phyla, while in the wild tomato rhizosphere mycobiome, the unclassified fungi (95.3%) were further enriched, whereas the relative abundance of the Ascomycota (4.5%) and Chytridiomycota (0.2%) was reduced as compared to the bulk soil (Fig. 3c). ASVs of abundant classified fungi in the rhizosphere were taxonomically delineated as *Fusarium* and *Aspergillus* (Fig. 3d).

## Functional diversity of the wild tomato rhizosphere bacteriome

**Fig. 4:**
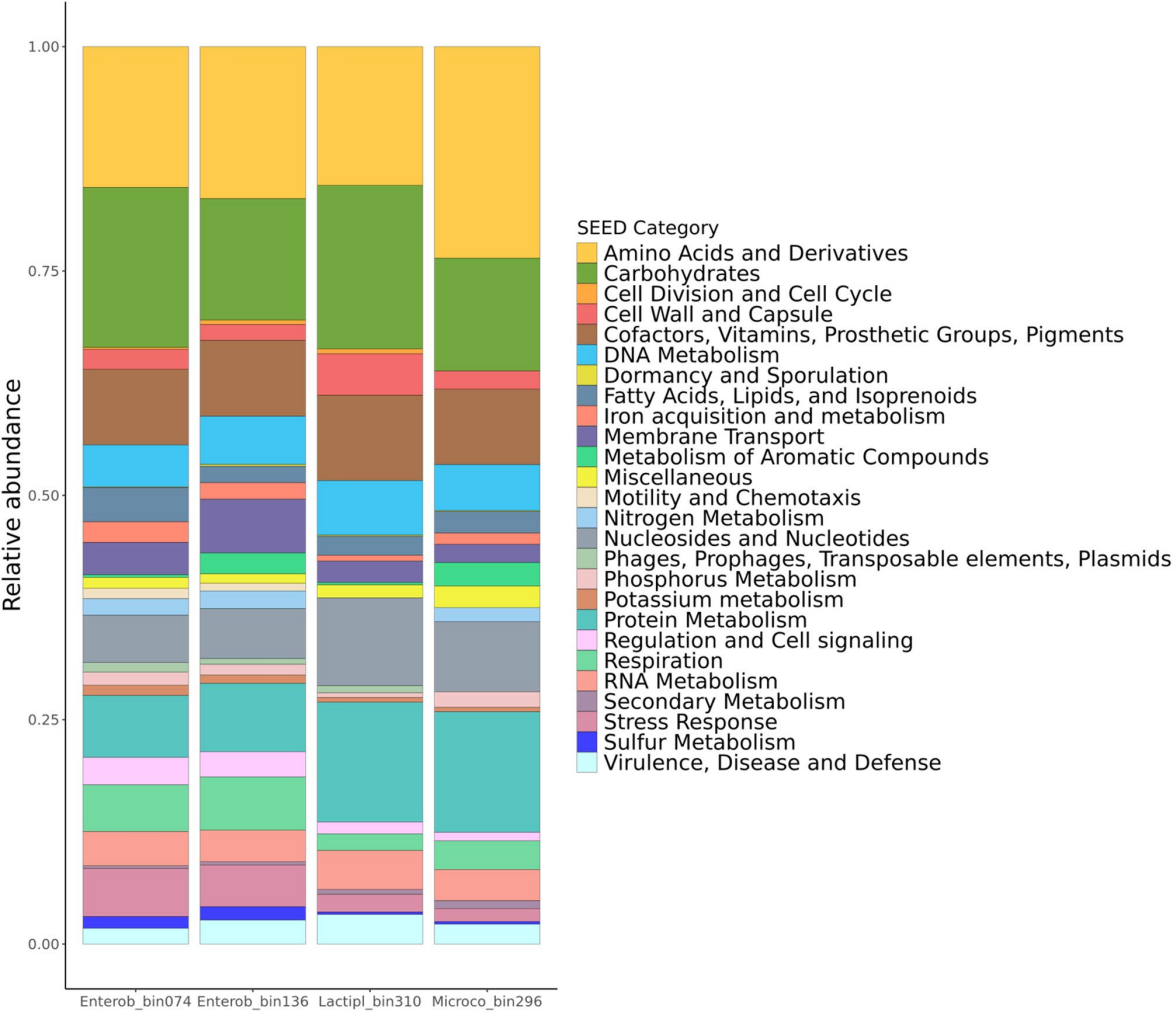
Annotation of bacterial metagenome-assembled genomes. Relative abundance of SEED categories annotated by RAST (Rapid Annotation using Subsystems Technology) from MAGs of Enterobacteriaceae (bins 074 and 136), *Lactiplantibacillus* (bin 310), and Micrococcaceae (bin 296) found in wild tomato rhizosphere. Protein encoding genes were grouped according their SEED categories and plotted as stacked bar charts.

Analyzing the significantly enriched rhizosphere bacterial ASVs for each sampling site, two ASVs were found to be conserved in the wild tomato rhizosphere across all three sites; these ASVs were taxonomically assigned as Enterobacteriaceae (ASV13; 2.41% relative abundance) and *Allorhizobium-Neorhizobium-Pararhizobium-Rhizobium* (ASV198; 0.12% relative abundance) (Supplementary Figure S8 and S9). To gain insights into the functional diversity of the wild tomato rhizosphere, particularly regarding these two ASVs, we conducted a shotgun sequence analysis of 24 rhizosphere DNA samples (7 from Calvas; 8 from Paltas and 9 from Zapotillo). This analysis resulted in four metagenome-assembled genomes (MAGs) belonging to the major rhizosphere enriched phyla in the 16 S amplicon data (Fig. 2c), two of them were assigned to the Enterobacteriaceae family (Proteobacteria, bin 074 and bin 136), one bin to the genus *Lactiplantibacillus* (Firmicutes, bin 310) and one to the Micrococcaceae (Actinobacteriota, bin 296) (Fig. 4). Unfortunately, no high-quality bins assigned as *Allorhizobium-Neorhizobium-Pararhizobium-Rhizobium* could be assembled (Supplementary Table S6).

Using the SEED subsystems database, the RAST server identified in the Enterobacteriaceae MAGs the highest number of protein-encoding genes (bin 074: 1307; bin 136: 1418), *Lactiplantibacillus* (bin 310: 757) and Micrococcaceae (bin 296: 1035 genes) (Supplementary Table S7). These four MAGs mostly included genes associated with SEED categories involved in the synthesis of amino acid and derivatives; carbohydrates; cofactors, vitamins, prosthetic groups, pigments; DNA metabolism; nucleosides and nucleotides; protein metabolism; RNA metabolism; virulence, diseases and defense (Fig. 4). In addition, the Enterobacteriaceae MAGs were characterized by a relatively high number of annotated genes associated with iron acquisition and metabolism, membrane transport, nitrogen metabolism, phosphorus metabolism, potassium metabolism, regulation and cell signaling, respiration, stress response, and sulfur metabolism. Furthermore, these MAGs harbored genes involved in motility and chemotaxis. *Lactiplantibacillus* bin 310 included mostly genes associated with cell wall and capsular polysaccharides and Micrococcaceae bin 296 included genes associated with metabolism or aromatic compounds and secondary metabolism (Table 1). Biosynthesis of the plant hormone auxin was a common secondary metabolism trait identified for all four MAGs representing the Enterobacteriaceae, *Lactiplantibacillus* and Micrococcaceae (Table 1).

**Table 1:** Main SEED subsystems found in MAGs of Enterobacteriaceae (bins 074 and 136), *Lactiplantibacillus* (bin 310), and Micrococcaceae (bin 296) associated with wild tomato *S. pimpinellifolium* rhizosphere.

Bacteria	Category	Subcategory	Subsystem	No. of genes
Enterobacteriaceae bin 074 and bin 136	Motility and Chemotaxis	Flagellar motility in Prokaryota	Flagellar motility	12
	Iron acquisition and metabolism	Iron acquisition and metabolism - no subcategory	Encapsulating protein for DyP-type peroxidase and ferritin-like protein oligomers	2
			Hemin transport system	5
		Siderophores	Siderophore Aerobactin	11
			Siderophore Enterobactin	14
	Membrane Transport	Protein secretion system, Type I	Type I secretion system for aggregation	4
		Protein and nucleoprotein secretion system, Type IV	IncF Conjugal Transfer System	21
			Conjugative transfer	21
			Type IV pilus	10
		Protein secretion system, Type V	Two partner secretion pathway (TPS)	2
		Protein secretion system, Type VII (Chaperone/Usher pathway, CU)	sigma-Fimbriae	5
			Type 1 pili (mannose-sensitive fimbriae, gamma-fimbriae)	2
	Stress Response	Osmotic stress	Osmoprotectant ABC transporter YehZYXW of Enterobacteriales	4
			Synthesis of osmoregulated periplasmic glucans	4
		Oxidative stress	Glutathione: Biosynthesis and gamma-glutamyl cycle	3
			Glutathione: Non-redox reactions	10
			Glutathione: Redox cycle	5
			Glutathionylspermidine and Trypanothione	2
	Sulfur Metabolism	Inorganic sulfur assimilation	Inorganic Sulfur Assimilation	16
		Organic sulfur assimilation	Alkanesulfonate assimilation	3
		Sulfur Metabolism - no subcategory	Galactosylceramide and Sulfatide metabolism	1
	Secondary Metabolism	Plant Hormones	Auxin biosynthesis	5
*Lactiplantibacillus* bin 310	Cell Wall and Capsule	Capsular and extracellular polysaccharides	Sialic Acid Metabolism	11
		Gram-Positive cell wall components	D-Alanyl Lipoteichoic Acid Biosynthesis	3
	Secondary Metabolism	Plant Hormones	Auxin biosynthesis	4
Micrococcaceae bin 296	Metabolism of Aromatic Compounds	Metabolism of central aromatic intermediates	Protocatechuate branch of beta-ketoadipate pathway	9
		Metabolism of central aromatic intermediates	Central meta-cleavage pathway of aromatic compound degradation	4
	Secondary Metabolism	Hydrocarbons	Alkane synthesis in bacteria 2	4
		Plant Hormones	Auxin biosynthesis	5

Next, mining of the MAGs for biosynthetic gene clusters (BGC) using antiSMASH revealed BGCs for carotenoids (terpene), dichrysobactin (NRP) and aerobactin (siderophore) in the Enterobacteriaceae MAGs. For the *Lactiplantibacillus* bin 310, seven BGCs were predicted with no or low similarities (< 50%) to known metabolites. Also, for Micrococcaceae bin 296, a BGC with 100% similarity to a carotenoid (terpene) BGC was found (Table 2; Supplementary Figure S10–S13).

**Table 2:** Biosynthetic gene clusters (BGCs) predicted by antismash bioinformatics tool from MAGs of Enterobacteriaceae (bins 074 and 136), *Lactiplantibacillus* (bin 310), and Micrococcaceae (bin 296) highly associated with wild tomato *S. pimpinellifolium* rhizosphere.

Bin ID	Length (nt)	antiSMASH type predictor	Secondary metabolite	Similarity	Reference in MiBIG database	Organism in MiBIG database
Enterobacteriaceae bin 074	23,579	Terpene	Carotenoid	100%	BGC0000642	Enterobacteriaceae bacterium DC413
Enterobacteriaceae bin 136	19,062	Terpene	Carotenoid	100%	BGC0000640	Enterobacteriaceae bacterium DC404
Enterobacteriaceae bin 136	14,409	NI-siderophore	Aerobactin	100%	BGC0001499	*Pantoea ananatis*
Enterobacteriaceae bin 136	39,218	NRP-metallopore, NRPS-like	Trichrysobactin, cyclic trichrysobactin, chrysobactin, dichrysobactin	84%	BGC0002414	*Dickeya chrysanthemi*
Enterobacteriaceae bin 136	43,855	NRPS-like	Minimycin	60%	BGC0002295	*Streptomyces hygroscopicus*
*Lactiplantibacillus* bin 310	26,778	NRPS	Mutanocyclin, leuvalin, tyrvalin	46%	BGC0002287	*Streptococcus mutans*
Micrococcaceae bin 296	17,892	Terpene	Carotenoid	100%	BGC0000633	*Streptomyces avermitilis*

## Discussion

To enhance our understanding of the diversity and microbial assembly in the rhizosphere of wild tomatoes in their center of origin in Ecuador, we analyzed the microbiome of bulk and rhizosphere soils from *S. pimpinellifolium* plants at flowering and fruiting developmental stage. These plants were growing naturally at three different sites in Loja province, southern Ecuador (Calvas, Paltas and Zapotillo). Our results showed different soil properties among these sites, with soils from Zapotillo displaying higher content of Fe, Mn, P, K, Cu, Zn and sand than soils from Calvas and Paltas. Wild *S. pimpinellifolium* typically grow in warm and dry climates of the Western Andes^12,13,24,25^. In Ecuador, *S. pimpinellifolium* occurs at high density in river valleys in semi-arid habitats of the ‘Low Andes’ region, where a natural depression in the Andes mountains occurs^11,26–28^. We sampled a total of 34 wild tomato individuals which were distributed mainly in places with temperatures around 30 ºC (Calvas: 29.8, Paltas 30.4, and Zapotillo 31.1 ºC) and dry environment with on average a relative humidity of only 7% (Calvas: 7.5, Paltas 6.6, and Zapotillo 6.1%) recorded during the field work.

The DArT genotyping results showed that the genetic diversity of the wild tomatoes corresponded to the sampling sites, suggesting limited spread of and gene flow between the wild tomato populations and adaptations to the abiotic factors prevalent in these geographically separated sites^8,29,30^. Particularly, climatic adaptation to temperature and precipitation has been proposed earlier for the genetic divergence of *S. pimpinellifolium* among regions^28,31^.

Microbiome analyses of the soils revealed that the bacterial and fungal community compositions were different among the native soil sites. Overall, soil bacteriomes mainly consisted of members of Firmicutes, Proteobacteria and Actinobacteriota, while soil mycobiomes included Ascomycota, Unclassified fungi and Basidiomycota. Similar microbiome composition profiles were described by Lee et al.^32^ in different locations of cultivated tomatoes in South Korea. However, we found that only bulk soil bacterial community variation correlated with soil physicochemical properties, especially with pH, CEC, clay, Ca, P, Mn and Fe (Fig. 1c). Soil texture provides microenvironments and determines water and nutrient retention^33^. For example, higher pH, CEC and clay content can explain a base saturation associated with higher soil fertility due to the readily available nutrients^34^. Furthermore, a correlation between soil texture and phosphorus content has been reported, where organic phosphorus forms were predominant in sandy soils and inorganic forms in finer textured soils^35^. With reference to nutrients, Ca content can alter the bacterial composition of the soil by increasing Firmicutes and Actinobacteriota and decreasing Gammaproteobacteria^36^as observed in the bulk soil from Paltas and Zapotillo (Supplementary Figure S6c).

Contrary to the soil bacteriome, the soil mycobiome diversity could not be explained by prevailing soil physicochemical properties. Previous studies have shown that edaphic parameters are poor predictors of the fungal diversity, whereas climatic variables such as aridity, precipitation, temperature, as well as plant cover or litter accumulation, are stronger predictors of fungal diversity^37–42^. Fungal phyla, such as Ascomycota, along with a significant proportion of unclassified fungi according to the UNITE database, were highly abundant in the native soils, including genera like *Alternaria*, *Albifimbria* and *Westerdykella* (Fig. 3d). The presence of a distinct group of ‘unclassified fungi’ highlights the limitations of current fungal databases and suggests that several fungal species in Southern Ecuador remain undiscovered or uncharacterized. Furthermore, this underscores our limited understanding of the soil mycobiome of this region, which represents an opportunity for future research. This composition as well as the high abundance of unidentified fungal taxa at phylum and genus levels were consistent with previous studies in arid environments^43,44^. Ascomycota phylum dominate soils globally, which is related with the versatile trophic capabilities for resource utilization, competition and stress tolerance^37^. For example, members of these phylum include dark septate fungi characterized by their melanin pigment, which allows survival in arid conditions and confers a competitive advantage over other fungi lacking these adaptations^45^. Basidiomycota is a diverse fungal group that includes mushrooms, smuts, rusts and yeasts, whose main contribution to the soil ecosystem is lignocellulose decomposition from wood and leaf litter^46,47^. Fungi belonging to the Chytridiomycota possess motile zoospores propelled by a posterior flagellum. Due to their smaller size, their rhizoid can attach to various substrates, including hard and resistant solids as sand or pollen grains. Additionally, Chytridiomycota efficiently digest cellulose, chitin and protein from soil organic matter and can respond to drought by forming desiccation-resistant sporangia as defense mechanisms^48,49^.

Rhizosphere bacteriome assembly of the wild tomato *S. pimpinellifolium* at flowering and fruiting developmental stages revealed not significantly different alpha and beta diversities among sampled sites. Moreover, the variation of the rhizobacterial composition could not be explained by the measured physicochemical soil properties nor by genotype diversity. These results suggest that *S. pimpinellifolium* as a host exerts selective pressure to orchestrate its bacterial community composition within its native habitat (R^2^ = 0.082; Fig. 2b), despite significant differences in soil types and soil bacteriomes. We further found that the persistent enriched bacteria ASVs in rhizosphere samples were Enterobacteriaceae and *Rhizobium* (Fig. 2d, Supplementary Figure S6e, S8 and S9). On the other hand, when we analyzed the samples from Paltas and Zapotillo separately, we discovered some interesting differences. Although the overall difference was only slightly significant, the variation in the rhizobacterial composition between these two sites could be attributed to the higher Ca content in the soil sampled from Paltas site (Supplementary Figure S7). This finding highlights the importance of site-specific environmental factors in microbial assembly. For example, Ca can modulate plant-microbe interactions and plant responses to abiotic stresses such as salt, drought, heat, and cold, by mediating signaling pathways that activate defense-related gene expression and phytohormone synthesis^50^. Additionally, an increase of Firmicutes and Actinobacteriota in response to higher Ca content has been observed in incubated soils with leaf litter^36^. Furthermore, the type and concentration of calcium salts has been identified as an important factor to enhance Actinobacteriota’s cultivation efficiency^51^.

Fungal communities associated with the wild tomato rhizosphere showed a similar pattern as observed for the rhizosphere bacteriome (Fig. 3c; Supplementary Figure S6d), displaying a group of fungi strongly associated with *S. pimpinellifolium* rhizosphere in its native habitat. Differential abundant ASVs assigned as *Fusarium* and *Aspergillus* genera were observed to be more abundant in the wild tomato rhizosphere (Fig. 3d; Supplementary Figure S6f). These genera have been abundantly identified also through culturing approaches in tomato rhizosphere^52,53^. The genus *Fusarium* includes well-known pathogenic species, which cause wilt, root necrosis or rot root in tomato^54^. During the sampling phase in the field, none of the sampled plants showed symptoms and no wilting was observed. Therefore, it is well possible that *Fusarium* found by amplicon sequencing represents nonpathogenic strains colonizing wild tomato rhizosphere. Nonpathogenic *Fusarium* strains regulate induced systemic resistance (ISR) genes and can elicit ethylene and nitric oxide production that stimulate formation of root hairs^55–58^. Also, *Aspergillus* has been found as a dominant fungus in the tomato rhizosphere^38,59,60^ and may serve as a plant growth promoter due to its capacity to produce hydrogen cyanide (HCN), indole-acetic acid (IAA) and siderophores, among other plant beneficial functions^61–63^. These results suggest that *S. pimpinellifolium* may also benefit from their native mycobiome, although isolation, genomic analyses and functional validation with cultured isolates will be needed to support these hypotheses.

In the metagenomic analysis, four high-quality bins were identified representing enriched bacterial genera from the predominant phyla found in the wild tomato rhizosphere, including Proteobacteria, Firmicutes and Actinobacteriota (Figs. 2c and 4; Supplementary Table S6). Analyses of the metagenome assembled genomes of the Enterobacteriaceae (bins 074 and 136) revealed functions related to motility and chemotaxis. These bacteria possess flagella for chemotaxis-oriented motility, which in turn enhances their ability to colonize roots. This motility advantage could make Enterobacteriaceae efficient rhizocompetitors^20,64–68^. Additionally, secretion systems found in Enterobacteriaceae bins may further enhance their colonization ability in tomato rhizosphere. For example, Type I and V secretion systems, are the ‘simplest’ secretion bacterial systems^69^. Type I transports proteins like toxins and adhesins directly from the cytoplasm to the extracellular environment, playing roles in biofilm formation and virulence^69–71^. While, Type V, particularly the Two-partner secretion system (TPS), facilitates adhesion to specific host cell structures through surface glycans^69,72–74^. As well as, Type IV are involved in the in the biogenesis of conjugative pili for DNA transfer and the delivery of effector proteins or toxins^75,76^whereas Type VII secretions systems are essential for bacterial adherence to surfaces, since these systems include Type 1 pili that bind to mannose residues on plant tissues^77–79^ Thus, Type IV and VII facilitate direct interactions between bacteria and host cells^80,81^.

For nutrient acquisition, Enterobacteriacea MAGs harbor several gene clusters involved in iron metabolism, including genes for encapsulins which are protein compartments that play roles in iron storage, oxidative stress resistance^82^and hemin transport systems for acquiring iron from organic sources^83,84^. Enterobacteriaceae bins also contained genes related to the production of siderophores, such as enterobactin and aerobactin, which chelate ferric iron (Fe³⁺) and enhance iron uptake^85,86^. Enterobactin, has a higher affinity for iron compared to aerobactin and pyoverdine, and therefore may increase competitiveness in interactions with neighboring rhizosphere microbes^20,87,88^. Enterobacteriaceae MAGs also showed key stress resistance features, such as osmotic stress resistance via osmoprotectant ABC transporters, such as YehZYXW, and the synthesis of osmoregulated periplasmic glucans, which help regulate intracellular osmolarity and maintain cell homeostasis^89–92^. Oxidative stress is mitigated by systems involved in glutathione metabolism, which are crucial for redox balance and protection against oxidative damage^93–95^. In addition, periplasmic stress responses in Enterobacteriaceae included genes that assist in the folding and degradation of outer-membrane proteins, essential for bacterial growth under high-temperature conditions^96,97^.

Only one high-quality metagenome assembled Firmicutes, another rhizosphere enriched lineage, was identified (Bin 310, representing *Lactiplantibacillus*, Supplementary Table S6). This *Lactiplantibacillus* MAG exhibited genes related to cell wall and capsule functions, specifically D-alanyl/lipoteichoic acid biosynthesis and sialic acid metabolism. Teichoic acids, comprising 30–70% of the Gram-positive cell wall, are vital for ion homeostasis, envelope assembly, flexibility and permeability, and aggregation^98–101^. Furthermore, D-alanylated teichoic acids in *Lactobacillus plantarum* optimize nutrient uptake through intestinal peptidase activity in *Drosophila*^102^. Sialic acid, found in bacterial capsules, may assist bacteria to water conservation and competitive adhesion ability in arid conditions^103–106^. As sialic acids are not present in plants or Archaea^106,107^it is likely *Lactiplantibacillus* may depend on other bacteria or fungi inhabiting the wild tomato rhizosphere as sources of this compound.

In the same way, bin 296, linked to Micrococcaceae, was the only one Actinobacteriota family MAG (Supplementary Table S6), harboring genes primarily involved in the metabolism of aromatic compounds through the protocatechuate branch and central meta-cleavage pathway. These pathways include aromatic compound and lignin degradation products like protocatechuic acid and catechol, which are crucial for energy, adaptation to suboptimal growth conditions, and protection against toxicity of aromatic compounds^108–111^. Additionally, bin 296 included genes for alkane synthesis from fatty acids, such as those coding for 3-oxoacyl-ACP synthase III and haloalkane dehalogenase-like proteins^112,113^. While the precise functions of alkane synthesis in bacteria is not fully understood, it is hypothesized that this process may contribute in protecting against temperature fluctuations or dehydration^112,114^.

It is noteworthy that all four analyzed MAGs harbored the genes for auxin biosynthesis, including anthranilate phosphoribosyltransferase, phosphoribosylanthranilate isomerase, and tryptophan synthase. Additionally, the presence of monoamine oxidase suggests involvement in tryptophan degradation for auxin synthesis^115,116^. Auxin, particularly indole-3-acetic acid (IAA), is critical for plant growth and development, influencing cell enlargement, tissue differentiation, and root modification, by promoting the formation of root hairs and lateral roots, which enhances nutrient and water uptake and overall plant health^117–121^.

In the context of secondary metabolism, selected MAGs associated with the wild tomato rhizosphere in its native habitat were analyzed using bacterial antiSMASH for biosynthetic gene clusters (BGCs) annotation (Table 2; Supplementary Figure S9–S12). BGCs are groups of genes that are physically clustered and encode pathways for the biosynthesis of specialized metabolites in bacteria, fungi, and plants^122^. Our analysis revealed that bins from Enterobacteriaceae and Micrococcaceae contain BGCs associated with carotenoid production. Carotenoids in soil and rhizosphere might can serve as precursors for abscisic acid (ABA), a key phytohormone involved in regulating water use during drought conditions and influencing root growth^123–125^. Additionally, carotenoids are known for their role in oxidative stress response, either by scavenging reactive oxygen species (ROS) or by reinforcing cell membranes to prevent oxidative damage^126^. Furthermore, non-ribosomal peptide synthase (NRPS) related BGCs were annotated in Enterobacteriaceae bin 136 and *Lactiplantibacillus* bin 310. In Enterobacteriaceae, we identified BGCs for trichrysobactin/cyclic trichrysobactin/chrysobactin/dichrysobactin and aerobactin. The production of these siderophores is critical for coping with fluctuations in iron availability^127–129^. The presence of multiple iron uptake systems provides a competitive advantage for colonizing hosts^127^. In *Lactiplantibacillus* bin 310, the NRPS-related BGCs with the highest similarity to known compounds were those associated with mutanocyclin/leuvalin/tyrvalin. Although the exact functions of these compounds remain unclear, they are hypothesized to play roles in host–microbiome interactions, potentially through regulation of electron transfer process and biofilm formation^130,131^.

This study provides a comprehensive analysis of the rhizosphere microbiome composition of wild tomato *S. pimpinellifolium* growing in its native habitat. Our findings revealed that despite variability among the wild tomato genotypes, physicochemical soil properties, and soil microbiomes, similar bacterial communities were assembled in the rhizosphere while exhibiting distinct fungal communities. The amplicon sequencing approach highlighted a strong and consistent association of Enterobacteriaceae and unknown fungi with the wild tomato rhizosphere across all sampled sites. This consistent predominance underscores the specialized roles of these microorganisms may play in the rhizosphere, potentially enhancing growth and resilience of the wild tomato plants in the harsh native environment. Furthermore, metagenomic analysis of bacterial bins from the Enterobacteriaceae family pinpointed specific features that may contribute to their robust association with the wild tomato rhizosphere. These features may facilitate the establishment of specialized functions that are crucial for the survival and functionality of the host–microbiome interaction in its native habitat. This aligns with findings that Enterobacteriaceae can thrive under water stress, potentially offering the plant enhanced resilience in dry areas^132–137^. These observations highlight the importance of regional studies in understanding plant-microbe interactions and generating various exciting hypotheses on the functional roles of specific microbial members of the microbiome of wild crop relatives grown in their native habitats. Isolation, genomic and extensive metabolic characterization of these conserved rhizobacterial taxa will be needed to allow functional validation of their role in growth and stress tolerance of wild tomatoes in their native habitats.

## Methods

### Fieldwork and sampling

**Fig. 5:**
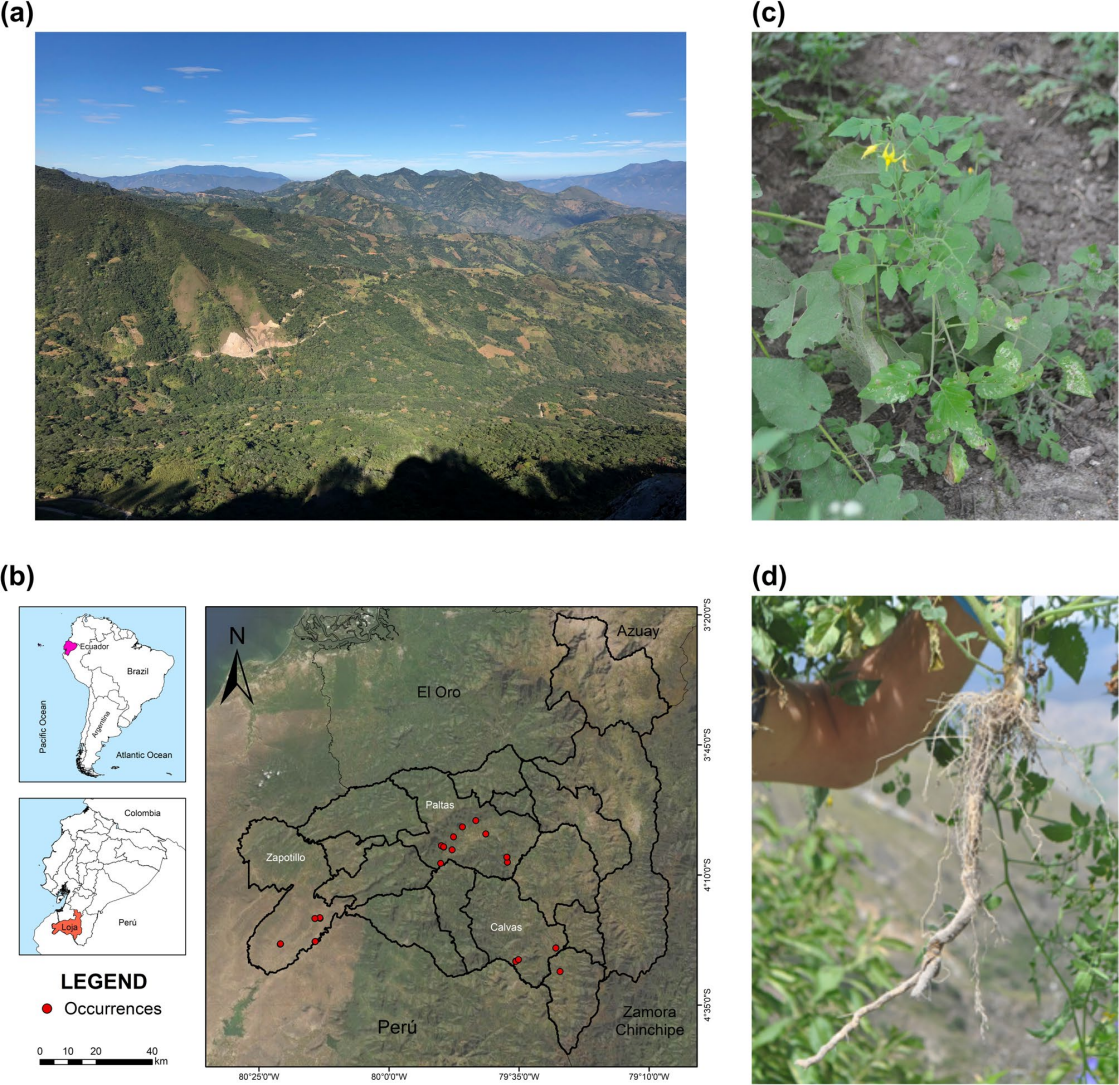
Sampling of wild tomato *Solanum pimpinellifolium* in their native habitat.** (a)** Landscape of native habitat of wild tomatoes in Loja, Ecuador; **(b)** Map of sampling sites of wild tomato native populations; **(c)** *S. pimpinellifolium* at flowering stage; **(d)** *S. pimpinellifolium* roots sampled for microbiome analysis.

A total of 34 plants of the wild tomato *S. pimpinellifolium* were collected in Loja province, southern Ecuador, where the most abundant population of wild tomatoes is found^138^; 8 from Calvas, 11 from Paltas, and 15 from Zapotillo. In total, 33 bulk soil samples, 34 rhizosphere soil samples and 34 leaf samples were collected. One bulk soil sample could not be taken due to the plant’s roots growing within rock fissures, making the soil inaccessible. Flowering and/or fruit-baring tomato plants of natural *S. pimpinellifolium* populations in the sites Calvas, Paltas and Zapotillo in the Loja province were sampled. The samples were found along the Calvas site, which had the highest altitude ranging from 1,365 to 1,196 m above sea level (masl), followed by Paltas which ranges from 1,434 to 666 masl and, Zapotillo, with the lowest altitude from 271 to 158 masl (Fig. 5; Supplementary Figure S1).

The sampling expedition of wild tomatoes was organized in June 2019, when plants were at flowering or fruiting stage. In the field, the areas for sampling were defined by natural barriers such as rivers and springs. The wild tomatoes were found growing at a variety of locations, for instance, next to fences, among farm crops (e.g. corn, common bean, cassava, intercropping), close to the rivers or springs, inside shrubby vegetation, etc. (Supplementary Figure S2–S4). Moreover, wild tomatoes were found with the help of local people living close to the selected sampling sites. Field samples were collected under sterile conditions using disposable gloves and 75% (v/v) ethanol. In addition, field site information was registered including geographic coordinates, altitude, local temperature, relative humidity in the shade, and the plant developmental stage.

For rhizosphere sampling, using a surface-sterilized steel digging bar, the soil around the tomato plant was dug up from 20 to 30 cm depth to uncover the root system. The roots were shaken vigorously to remove the loose soil particles as much as possible. Roots with tightly attached soil were cut into 2–3 cm segments and collected into a 50 ml-tube, mixed with 4 ml of LifeGuard^®^ Soil Preservation Solution (Qiagen, USA). Samples were stored in a cooler box with ice gel blocks for transportation to the laboratory. For bulk soil sampling, 4 g of soil were collected at 1 m distance from the sampled individual with no visual presence of roots, and placed into a 15 ml-tube and mixed with 4 ml of LifeGuard^®^ Soil Preservation Solution. After rhizosphere sampling, 1 kg of the soil from the same place where the plant grew was collected in a zip-lock bag for physicochemical analysis. Furthermore, three fully expanded young leaves were cut with 75% ethanol-sterilized scissors from the top of each sampled plant and placed into a paper bag for later plant genotyping at Diversity Arrays Technology Pty Ltd (Bruce, Australia).

### Soil physicochemical analysis

Back at the laboratory, 33 soil samples were air dried and sieved (2 mm diameter sieve mesh) and sent to Agrocalidad Laboratory facilities (Tumbaco, Ecuador) for standard physicochemical analysis. Soil physicochemical data were normalized by log transformation [log10(x + 1)] to calculate their Euclidean distance and perform permutational analysis of variance (PERMANOVA, 9,999 permutations *p* < 0.05) using vegan R package^139^ among sites, and perform a principal components analysis (PCA, *prcomp* function).

### Soil and rhizosphere DNA isolation and amplicon and metagenome sequencing

The rhizosphere samples were pre-processed by pulsing the vortex at maximum speed multiple times, sequentially, to effectively dislodge and transfer as much soil as possible from the roots into the LifeGuard^®^ Soil Preservation Solution. Then the roots were removed from the tubes and the rhizosphere soil suspension was transferred by pipetting with a cut pipette tip into 15 ml-tubes and stored at −20 ºC until DNA extraction. The rhizosphere and bulk soil samples were prepared by pelleting 0.5 g of soil by centrifuging aliquots (1.8 ml) of soil suspension at 10,000 × *g* for 1 min, and discarding the supernatant of LifeGuard^®^ Soil Preservation Solution. The Qiagen DNeasy^®^ PowerSoil^®^ Kit was used to isolate the genomic DNA according to the manufacture’s kit protocol. DNA samples were sent to BaseClear (Leiden, The Netherlands) for amplicon library preparation and subsequent sequencing of the V3-V4 regions of the 16 S rRNA gene using the universal bacterial primers 341 F (CCTACGGGNGGCWGCAG) and 805R (GACTACHVGGGTATCTAATCC), while the primers ITS3F (GCATCGATGAAGAACGCAGC) and ITS4R (TCCTCCGCTTATTGATATGC) were used to sequence the ITS2 region. Paired-end sequence reads (2 × 250 bp) were generated using the Illumina MiSeq platform, performed under accreditation according to the scope of BaseClear B.V. (L457; NEN-EN-ISO/IEC 17025). Shotgun sequencing was performed on 24 rhizosphere DNA samples to generate paired-end sequences with the length of 150 bp per read using the NovaSeq platform according to the scope of BaseClear B.V. (L457; NEN-EN-ISO/IEC 17025).

### Plant DNA isolation and DArT-SNP genotyping

Dry leaflet samples of 34 individuals of the native tomato populations were used for genotyping. Three seed bank accessions of *S. pimpinellifolium* (CGN14498 and CGN23957, identified as LPI and SPI, respectively) and the domesticated tomato *S. lycopersicum* cv. Moneymaker (CGN14330, identified as MON) were included for comparative purpose (Supplementary Table S1). Dry leaflets were pulverized using the Qiagen TissueLyser II Bead Mill and 50 mg of leaf powder was send to Diversity Arrays Technology (DArT, Bruce, Australia) for plant DNA extraction and tomato genotyping using the DArTseq service. The presence/absence data of 12,745 single-nucleotide polymorphisms (SNPs) from the 34 tomato samples, two *S. pimpinellifolium* accessions and one *S. lycopersicum* cv. Moneymaker (37 tomato genotypes in total) were used to perform the hierarchical clustering using the Jaccard distance in the vegan package; a dendrogram plot was generated using ape package^140^. For the permutational analysis of variance (PERMANOVA, 9,999 permutations *p* < 0.05), 11,923 SNPs from 34 tomato samples were used in the vegan R package^139^ to analyze the genetic diversity among sites.

### Amplicon sequence analysis

The compressed sequence reads in FASTQ format were processed by the DADA2 v1.16.0 pipeline^141^ in RStudio environment^142^ to obtain the abundance and taxonomy tables. The taxonomy assignment for bacteria was performed with the SILVA ribosomal RNA gene reference database (v138)^143^ while the UNITE database (version 8.2) was used for fungal taxonomy assignment^144^.

A total of 1,744,884 of high-quality bacterial sequences were obtained, with a mean of 22,660 sequences per sample. For fungi, a total of 2,378,471 sequences were obtained, averaging 30,889 sequences per sample (Supplementary Table S2). ASVs assigned to Archaea, Mitochondria, Chloroplast and non-fungal Eukaryota were removed prior to downstream analyses. Finally, after taxonomic assignment, 18,452 ASVs were identified as bacterial and 14,241 as fungal ASVs. The statistical analysis was performed in RStudio environment and R software version 4.3.1^145^. R packages such as tidyverse^146^ vegan^139^ phyloseq^147^, metagenomeSeq^148^ and ggplot2^149^ were used for alpha diversity (ANOVA, Tukey HSD post hoc test), beta diversity (Bray–Curtis distance, PERMANOVA with 9,999 permutations), and differential abundance analyses. The abundance data were normalized by CSS (Cumulative Sum Scaling) before all analyses. To examine the relationship between wild tomato genetic diversity (Jaccard) and microbiome (Bray–Curtis) distance, as well as between physicochemical soil properties (Euclidean) distance and microbiome (Bray–Curtis) distance, both in bacterial and fungal communities, Mantel tests were performed on Spearman correlations. Moreover, the function *envfit* from the vegan package was used to determine the physicochemical soil properties related to the microbiome distribution (bacteria or fungi). Principal Coordinates Analysis (PCoA) was done with the vegan package using the *cmdscale* function and the Bray–Curtis distance calculated previously. Differential abundance analysis was performed using the metagenomeSeq package. Microbiome data (ASV abundances) were normalized using CSS normalization. Low-abundance ASVs were filtered based on the effective sample size with the *calculateEffectiveSamples* function. The model “~Soil_type” was defined to test for differential abundance of ASVs between soil types (bulk soil vs. rhizosphere) using the *fitZig* function. After identifying differentially abundant ASVs, a new dataset was created containing those ASVs with significant log(2) fold change (adjusted *p* < 0.05). Additionally, the taxonomy of these ASVs was included for further interpretation and result visualization. This procedure was also applied to determine the shared rhizosphere bacterial ASVs between sites. In this case, bulk soil and rhizosphere data were analyzed by site, and significantly abundant ASVs in each rhizosphere were separated into a new dataset for comparison. An UpSet plot to determine the ASVs shared between sites was generated by UpSetR software^150^.

### Metagenome data analysis

The paired-end sequence read libraries in FASTQ format were processed using SqueezeMeta v1.5.1^151^. Co-assembly was done using Megahit^152^. Contig statistics were calculated using PRINSEQ^153^ then redundant contigs were removed using CD-HIT^154^ and contigs were merged using Minimus2^155^. Furthermore, RNAs were predicted using Barrnap^156^ and 16 S rRNA sequences were taxonomically classified using the RDP classifier^157^. tRNA/tmRNA sequences were predicted using ARAGORN^158^while ORFs were predicted using Prodigal^159^. Similarity searches for GenBank^160^ EggNOG^161^ and KEGG^162^ were done using Diamond^163^. Read mapping against contigs was performed using Bowtie2^164^. Additionally, binning was done using MaxBin2^165^ and Metabat2^166^. These binning results were combined using DAS Tool^167^ to obtain refined bins.

Relevant information from the metagenomics data (ORF, contig and bin annotations, aggregated taxonomic and functional features) was exported into tables by running the SqueezeMeta utility script sqm2tables.py^151^ to facilitate data handling for further analyses.

Genome bins were qualitatively assessed by CheckM^168^. Afterwards, two high quality bin files belonging to the Enterobacteriaceae family, one to *Lactiplantibacillus* and one to Micrococcaceae were selected and submitted to the RAST server (Rapid Annotation using Subsystems Technology)^169^ to annotate their functional genes. Also, the bin files were submitted to the bacterial antiSMASH software (Antibiotics & Secondary Metabolite Analysis Shell) version 7^170^ to identify biosynthetic gene clusters and predicted metabolites.

## Electronic supplementary material

Below is the link to the electronic supplementary material.

Supplementary Material 1

Supplementary Material 2

## Data Availability

The 16 S and ITS amplicons and shotgun metagenomics sequencing data have been deposited in the European Nucleotide Archive (ENA) database under the project accession number PRJEB82447. Further inquiries can be directed to the corresponding author Pieter van ’t Hof at Universidad San Francisco de Quito (pvanthof@usfq.edu.ec).

## Declarations

### Plant material collection permits

This study was carried out under the Genetic Resource Permit numbers **MAE-DNB-CM-2018-0085** and **MAATE-DNB-CM-2022-0271**, issued by the Ecuadorian Ministry of Environment to USFQ. During field sampling, we collected only the minimum number of *S. pimpinellifolium* specimens necessary to achieve the objectives of this research. We prioritized the collection of post-reproductive individuals (flowering and/or fruit-baring tomato plants) to help preserve natural wild tomato populations at the sampling sites. The formal identification of the plant material was carried out in situ by Stalin Sarango Flores and Ben O. Oyserman. No voucher specimens were collected.
