# Supplementary material for: Unveiling diversity and adaptations of the wild tomato Microbiome in their center of origin in the Ecuadorian Andes
Source: Sci Rep. 2025 Jul 1;15:22448. doi: 10.1038/s41598-025-05816-1 (PMC12215834; doi:10.1038/s41598-025-05816-1)
Supplement: Supplementary file 2 — Supplementary Material 2 [file 41598_2025_5816_MOESM2_ESM.pdf]

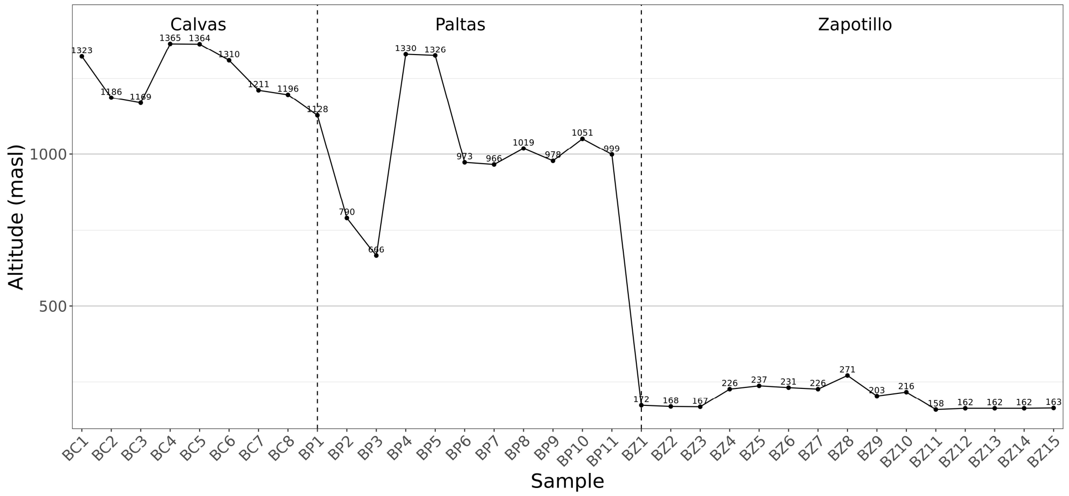

Figure S1. Elevation in meters above sea level (masl) of the wild tomato sites sampled in Loja, Ecuador.

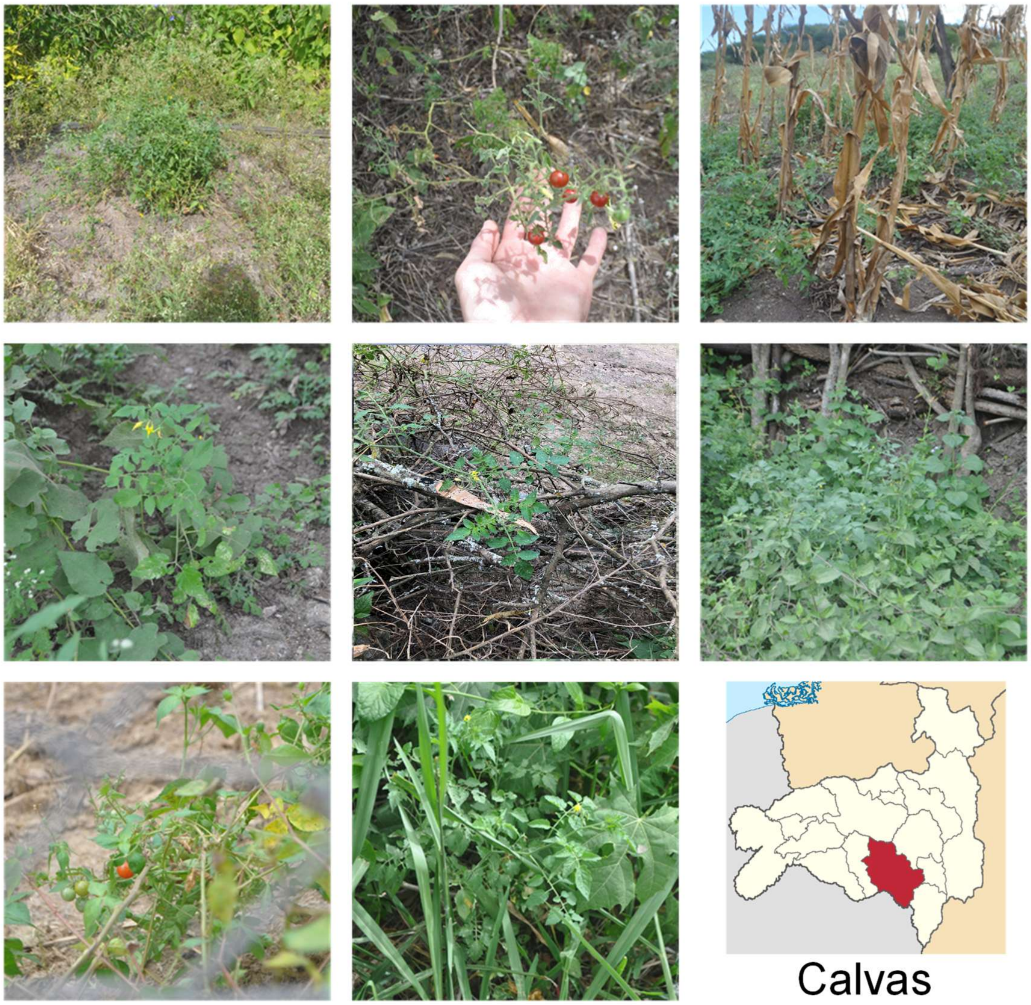

Figure S2. Eight *S. pimpinellifolium* sampled in Calvas (Loja, Ecuador).

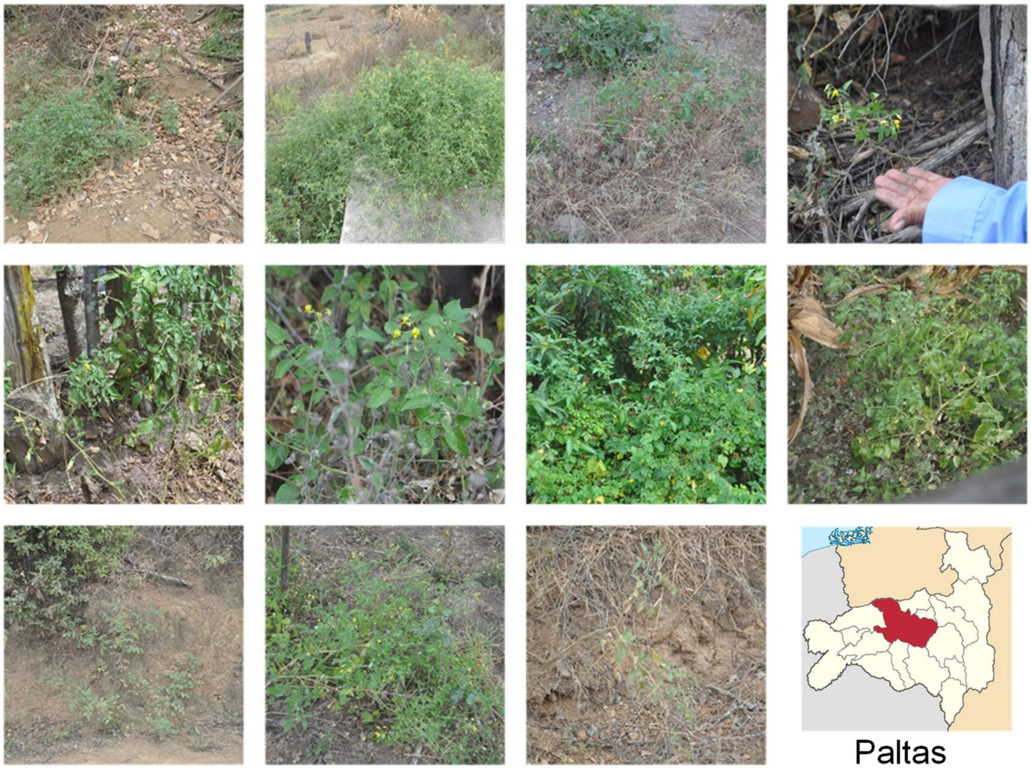

Figure S3. Eleven *S. pimpinellifolium* sampled in Paltas (Loja, Ecuador).

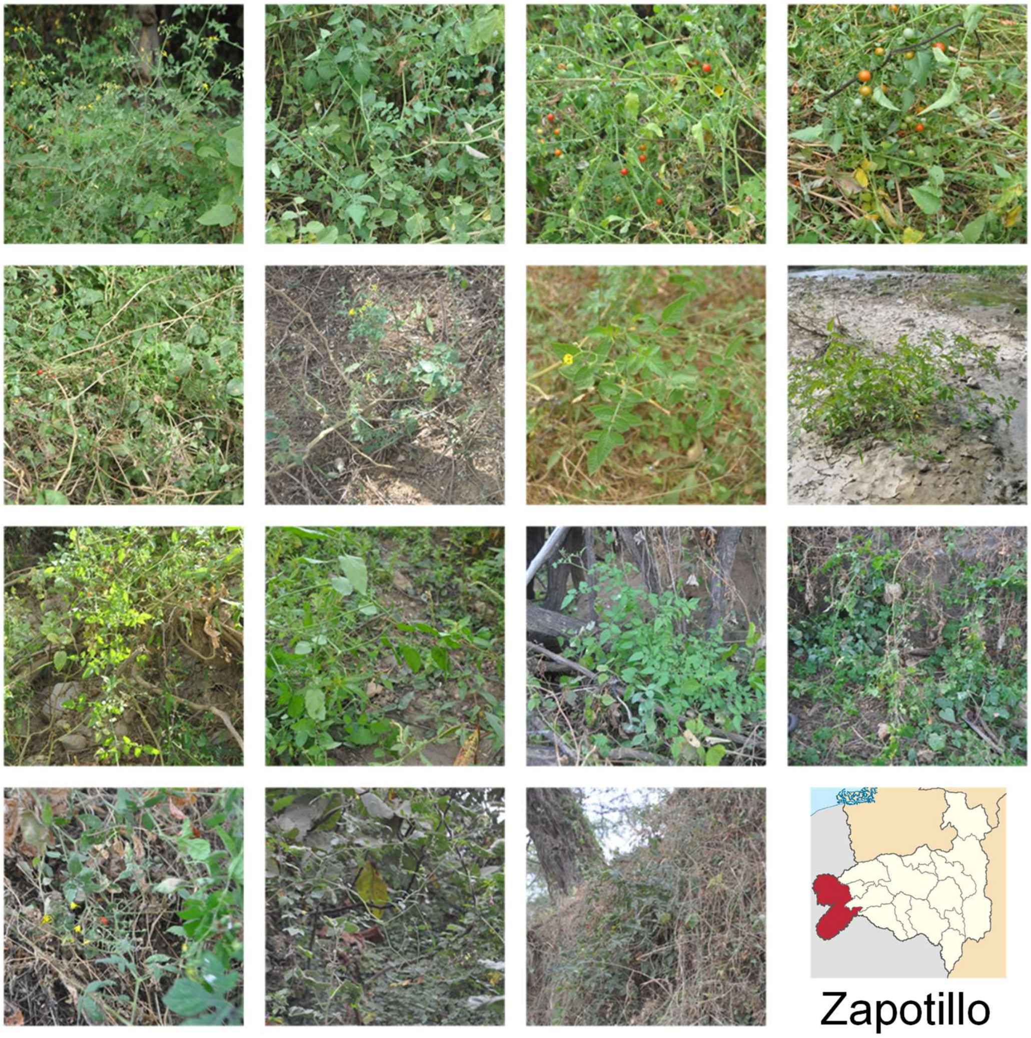

Figure S4. Fifteen *S. pimpinellifolium* sampled in Zapotillo (Loja, Ecuador).

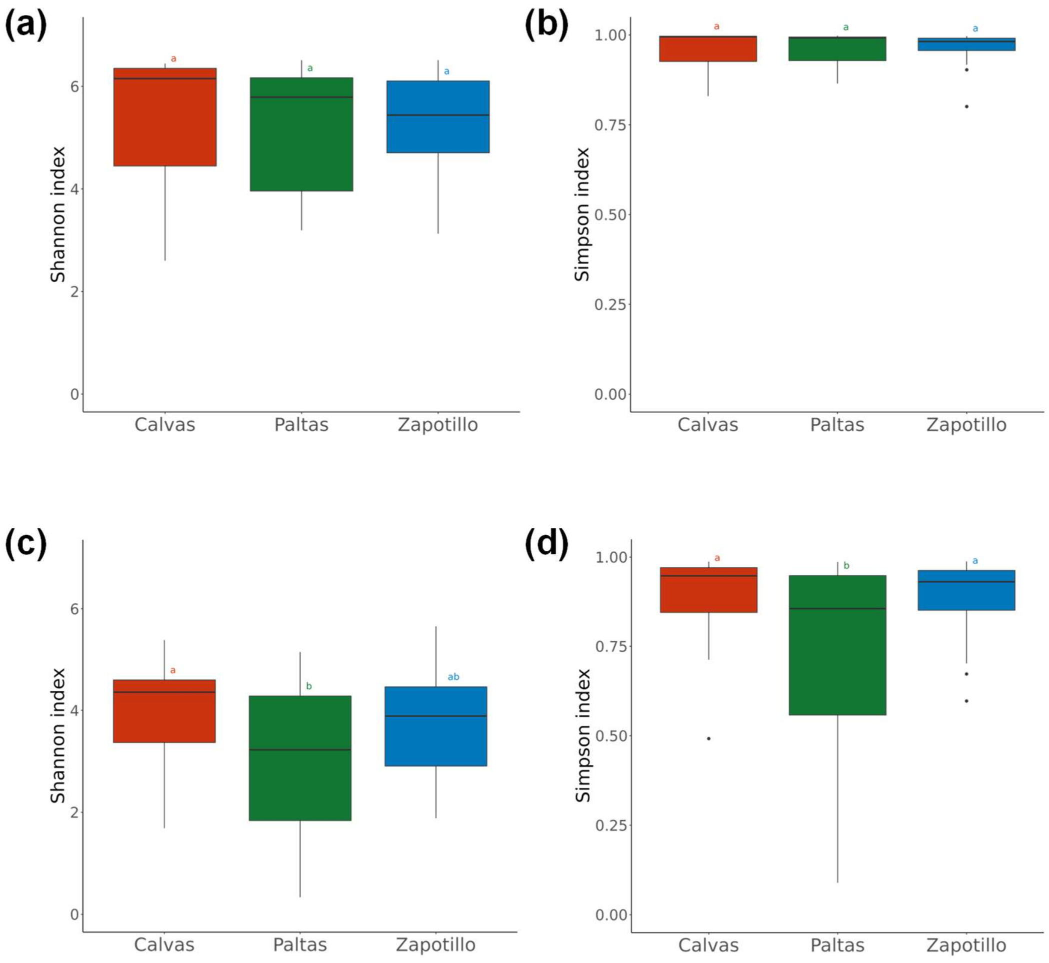

Figure S5. Alpha diversity in bulk soil samples. Shannon **(a)** and Simpson diversity index **(b)** of bacterial communities. Shannon **(c)** and Simpson diversity index **(d)** of fungal communities.

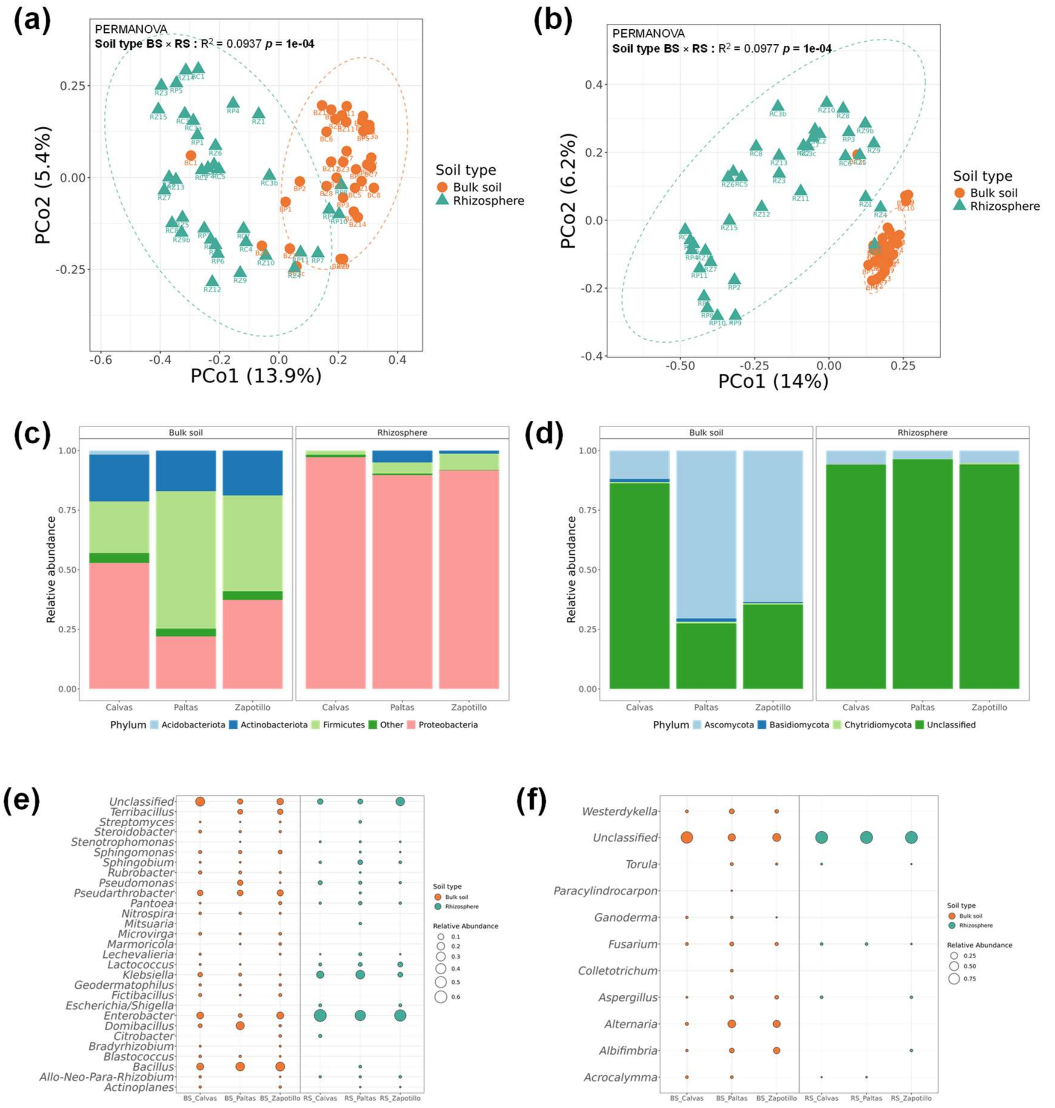

Figure S6. PCoA of **(a)** bacterial and **(b)** fungal communities of bulk soil and rhizosphere samples of *S. pimpinellifolium* in its native habitat; profile of bacteria. Relative abundance of bacterial **(c)** and fungal **(d)** phyla in bulk and rhizosphere soil of wild tomato *S. pimpinellifolium* in three sites of its native habitat. Relative abundance of bacterial **(e)** and fungal **(f)** genera in bulk and rhizosphere soil of wild tomato *S. pimpinellifolium* in three sites of its native habitat (Loja, Ecuador).

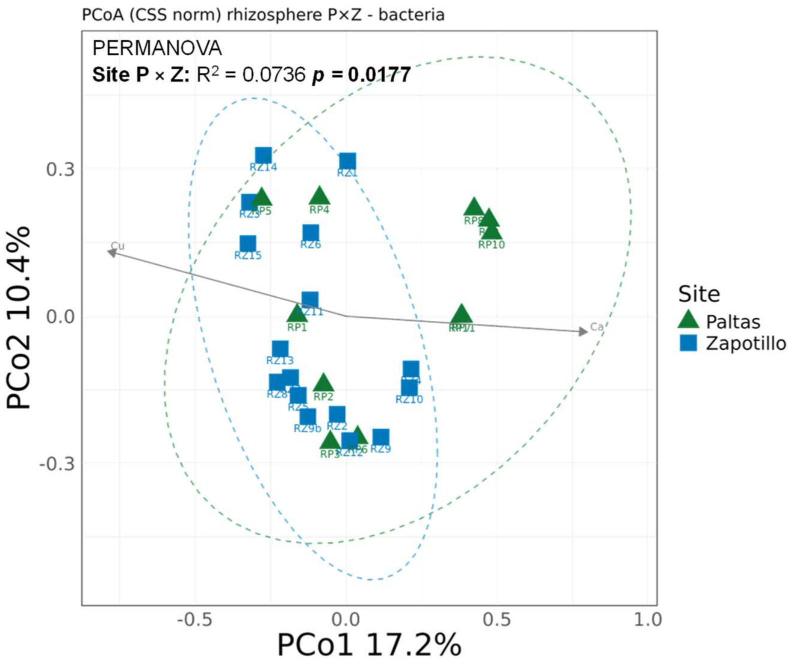

Figure S7. PCoA of rhizosphere bacterial communities from Paltas and Zapotillo samples with significant related soil properties.

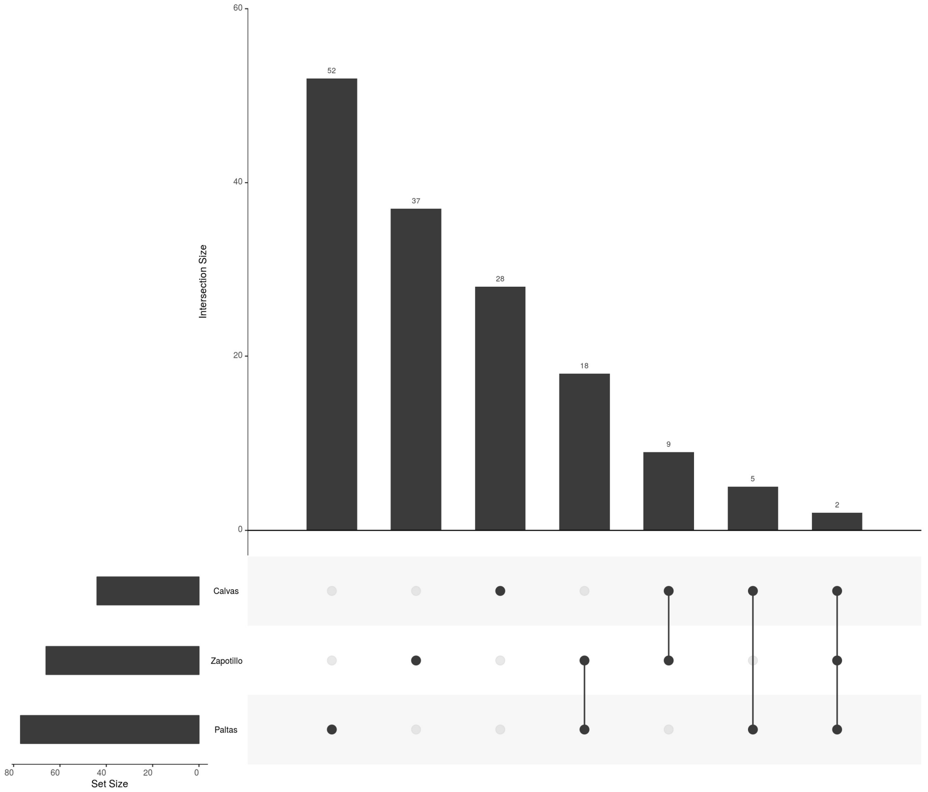

Figure S8. Number of bacterial ASVs shared among wild tomato rhizosphere samples (upset plot generated by UpSetR software (Lex et al., 2014)).

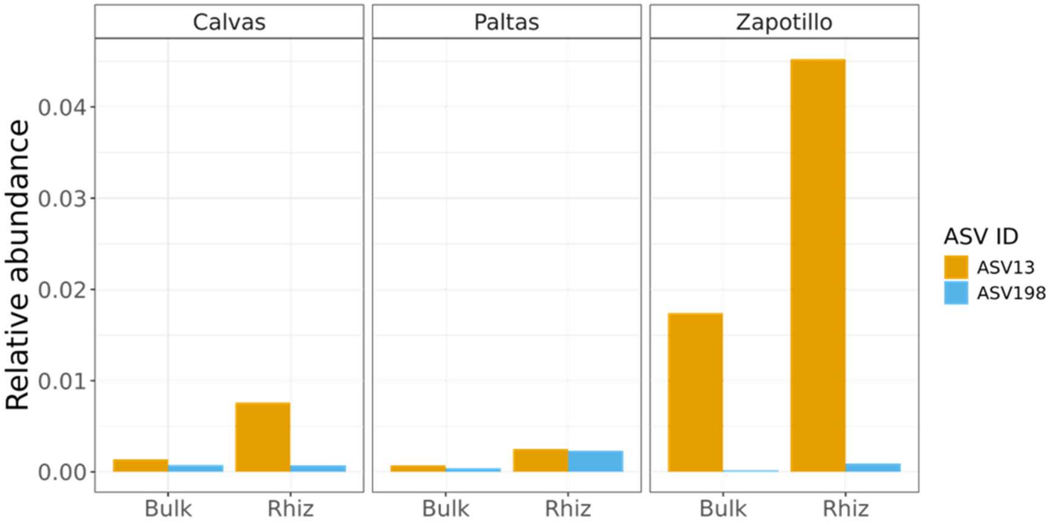

Figure S9. Relative abundance of the two ASVs (ASV13 and ASV198) shared among wild tomato rhizosphere samples.

Overview 4.1 15.1 35.1 37.1 43.1 59.1

Identified secondary metabolite regions using strictness 'relaxed'

Region	Type	From	To	Most similar known cluster		Similarity
Region 4.1	redox-cofactor	1	9,031	lankacidin C	NRP+Polyketide	13%
Region 15.1	terpene	22,928	46,506	carotenoid	Terpene	100%
Region 35.1	RRE-containing	135,528	155,450	lankacidin C	NRP+Polyketide	13%
Region 37.1	NRPS	16,593	88,392	FR900359	NRP:Cyclic depsipeptide	25%
Region 43.1	NRPS	41,285	65,468			
Region 59.1	RiPP-like	1	8,547			

Figure S10. Overview of BGCs found in Enterobacteriacea bin 074 by bacterial antiSMASH software (Blin et al., 2023).

Overview 82.1 87.1 93.1 98.1 101.1 101.2 134.1

Identified secondary metabolite regions using strictness 'relaxed'

Region	Type	From	To	Most similar known cluster		Similarity
Region 82.1	terpene	1	19,062	carotenoid	Terpene	100%
Region 87.1	thiopeptide	114,457	140,767	O-antigen	Saccharide	14%
Region 93.1	NRP-metallophore, NRPS	26,276	65,493	trichrysobactin/cyclic trichrysobactin/chrysobactin /dichrysobactin	NRP	84%
Region 98.1	NRPS-like	59,101	102,955	minimycin	NRP+Saccharide	60%
Region 101.1	NI-siderophore	72,134	86,542	aerobactin	Other	100%
Region 101.2	phosphonate	88,908	100,218			
Region 134.1	ladderane, arylpolyene	11,070	54,850			

Figure S11. Overview of BGCs found in Enterobacteriacea bin 136 by antiSMASH software (Blin et al., 2023).

Overview | 12.1 | 19.1 | 24.1 | 26.1 | 33.1 | 37.1 | 51.1

Identified secondary metabolite regions using strictness 'relaxed'

Region	Type	From	To	Most similar known cluster		Similarity
Region 12.1	T3PKS	511,783	552,952			
Region 19.1	NRPS	11,746	38,523	mutanocyclin/leuvalin/tyrvalin	NRP	46%
Region 24.1	cyclic-lactone-autoinducer	114,616	135,321			
Region 26.1	RiPP-like	22,938	35,088			
Region 33.1	NRPS, NRPS-like	9,760	69,443			
Region 37.1	terpene	955	20,601			
Region 51.1	NRPS	1	11,926	mutanocyclin/leuvalin/tyrvalin	NRP	30%

Figure S12. Overview of BGCs found in *Lactiplantibacillus* bin 310 by antiSMASH software (Blin et al., 2023).

Overview | 6.1 | 13.1 | 29.1 | 33.1 | 54.1

Identified secondary metabolite regions using strictness 'relaxed'

Region	Type	From	To	Most similar known cluster		Similarity
Region 6.1	terpene	64,916	85,854	carotenoid	Terpene	37%
Region 13.1	terpene	1	17,892	carotenoid	Terpene	100%
Region 29.1	betalactone	368	26,286	microansamycin	Polyketide	7%
Region 33.1	NAPAA	1,207	35,076	stenothricin	NRP:Cyclic depsipeptide	27%
Region 54.1	RiPP-like	1	6,403			

Figure S13. Overview of BGCs found in Micrococcaceae bin 296 by antiSMASH software (Blin et al., 2023).
